# Hidden in the crowd: primordial germ cells and somatic stem cells in the mesodermal posterior growth zone of the polychaete *Platynereis dumerillii *are two distinct cell populations

**DOI:** 10.1186/2041-9139-3-9

**Published:** 2012-04-18

**Authors:** Nicole Rebscher, Anika Kristin Lidke, Christian Friedrich Ackermann

**Affiliations:** 1 Morphology and Evolution of Invertebrates, Philipps-Universität Marburg, Karl von Frisch Strasse 8, 35032 Marburg, Germany; 2 Nikon Imaging Center, 69120 Heidelberg, Germany

## Abstract

**Background:**

In the polychaete *Platynereis*, the primordial germ cells (PGCs) emerge from the *vasa*, *piwi*, and *PL10 *expressing mesodermal posterior growth zone (MPGZ) at the end of larval development, suggesting a post-embryonic formation from stem cells.

**Methods:**

In order to verify this hypothesis, embryos and larvae were pulse labeled with the proliferation marker 5-ethynyl-2'-deoxyuridine (EdU) at different stages of development. Subsequently, the PGCs were visualized in 7-day-old young worms using antibodies against the Vasa protein.

**Results:**

Surprisingly, the primordial germ cells of *Platynereis *incorporate EdU only shortly before gastrulation (6-8 hours post fertilization (hpf)), which coincides with the emergence of four small blastomeres from the mesoblast lineage. We conclude that these so-called 'secondary mesoblast cells' constitute the definitive PGCs in *Platynereis*. In contrast, the cells of the MPGZ incorporate EdU only from the pre-trochophore stage onward (14 hpf).

**Conclusion:**

While PGCs and the cells of the MPGZ in *Platynereis *are indistinguishable in morphology and both express the germline markers *vasa*, *nanos*, and *piwi*, a distinct cluster of PGCs is detectable anterior of the MPGZ following EdU pulse-labeling. Indeed the PGCs form independently from the stem cells of the MPGZ prior to gastrulation. Our data suggest an early PGC formation in the polychaete by preformation rather than by epigenesis.

## Background

In many species, ranging from sponges, and cnidarians, to flatworms, annelids, tunicates, and sea urchins, both primordial germ cells (PGCs) and somatic stem cells are characterized by the expression of a similar set of genes, namely *vasa*, *nanos*, *piwi*, and *PL 10 *[1-7]. This fact led to the hypothesis that PGCs and stem cells are not only closely related, but also share a common gene regulatory module, the 'germline multipotency program' [8,9]. Numerous examples show that pluripotent stem cells give rise to both PGCs and somatic derivates, such as choanocytes in sponges, large I-cells in cnidarians, neoblasts in planarians, or epiblast cells in mice [3,7,10,11]. The generation of PGCs by undifferentiated cells occurs after gastrulation and sometimes is referred to as 'epigenesis' [12]. It requires inductive signals by the surrounding tissue. In mice, for example, BMP 4 and 8 secreted by the extra-embryonic ecto-and endoderm induce the expression of germ cell specific genes in cells of the proximal epiblast [13]. In other species, the PGCs form early in development self-autonomously by inheritance of cytoplasmic determinants contained in ribonucleotide particles of the germ plasm, which has been termed 'preformation' [12]. Epigenesis is currently considered to constitute the ancestral mechanism, while preformation might have arisen in evolution several times independently as an adaptation to fast embryogenesis and/or extensive reorganization during gastrulation [14,15].

A recent finding in the cephalochordate *Branchiostoma*, however, challenges the model of a stem cell origin of PGCs: in this species, the PGCs had for a long time been considered to arise post-embryonically from the stem cells of the posterior growth zone by epigenesis [15], but a closer examination now revealed that they are in fact specified during early cleavage stages by inheritance of a maternal *vasa *and *nanos *containing germ plasm, that is by preformation [16]. Later, a second *vasa *and *nanos *expressing domain forms around the blastopore during gastrulation, which harbors the future somatic stem cells of the posterior growth zone. The PGCs then move from the ventral endoderm towards this region and blend into this cell mass, which they again leave as single cells in the late neurula stage [16]. Thus, although the cells of the posterior growth zone in the early neurula stage are indistinguishable by morphology and both co-express *vasa *and *nanos*, they, in fact, constitute a patchwork of PGCs and somatic stem cells.

**Figure 1:**
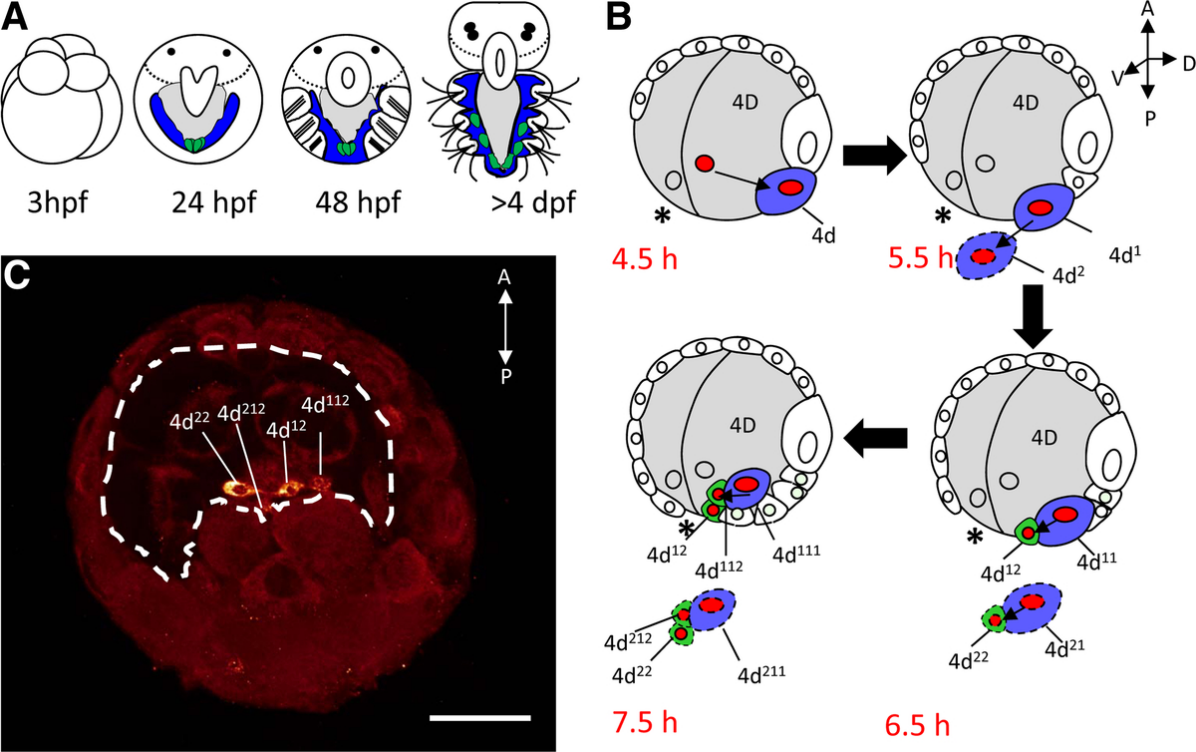
**Development of the mesoblast 4d**. (**A**) Developmental stages of *Playtnereis dumerilli*: the embryo undergoes spiral cleavage and develops into a free swimming trochophore larva within 24 hours post fertilization (hpf). A cluster of *vasa*-positive cells is detectable at the base of the mesodermal bands. One day later, the anlagen of the first three segments as well as the stomodaeum are detectable in the metatrochophore larva. The nectochaete larva (72 hpf) exhibits three parapodial segments with bristles. The four PGCs leave the MPGZ in young worms of 96 hpf. Blue: mesodermal bands, green: PGCs and MPGZ, grey: gut. (**B**) Formation of the primordial germ cells, same colors are used as in (A), lateral view, modified after [18]; figures 72 and 73. The animal pole, defined by the position of the polar bodies and later by the apical organ, is pointing to the top. The blastopore (asterisk) lies at the future ventral side. The MPGZ demarks the posterior pole, while the somatoblast lies on the dorsal side. Embryos are drawn as optical sections in lateral orientation with anterior to the top and dorsal to the right. Only the right half of the embryo is shown in detail, the corresponding cells on the left side are shown with dashed lines. During epibolic gastrulation, the micromeres overgrow the macromeres, the future endoderm. Around 4.5 hpf, the mesoblast 4d buds of from the macromere 3D at the ventral ridge of the closing blastopore (asterisk). The mesoblast divides bilaterally around 5.5 hpf, giving rise to the two primary mesoblasts 4d^1 ^and 4d^2^. At 6.5 hpf, the right mesoblast buds off the small secondary mesoblast cell 4d^12 ^towards the blastopore. A second small blastomere (4d^22^) arises simultaneously from the left mesoblast (dashed lines). At 7.5 hpf, the primary mesoblasts give rise to a second pair of secondary mesoblasts, 4d^112 ^and 4d^212^. (**C**) Embryo at 10 hpf, exhibiting the four Vasa-positive secondary mesoblast cells at the posterior rim of the blastopore (dashed line). Ventral view on the blastopore, anterior is to the top. Scale bar = 50 μm.

In order to investigate whether such a mosaic of PGCs and somatic stem cells also exists in other phyla, PGC formation in the polychaete *Platynereis *was subjected to a re-examination. Here, a two-step mechanism had previously been proposed, consisting of an early determination of the mesodermal posterior growth zone (MPGZ) by cytoplasmic determinants (preformation) and a postembryonic formation of the PGCs from the MPGZ by epigenesis [1]. As in other species, both the PGCs and the somatic stem cells of the MPGZ in *Platynereis *express the typical 'germ cell regulatory network' *vasa*, *nanos*, *PL10*, and *piwi*. Four PGCs emerge from the MPGZ at 96 hours post fertilization (hpf), thus at the end of larval development (Figure 1A). In the polychaete *Capitella*, PGCs are similarly first detectable as two *vasa *and *nanos *expressing cell clusters in late larval stages [17]. However, in this species, *vasa *and *nanos *are already up-regulated in descendants of the mesoblast 4d prior to gastrulation. In the *Platynereis *embryo, four so-called secondary mesoblast cells have been observed previously to bud from the mesoblast descendants 4d^1 ^and 4d^2 ^around the onset of gastrulation. [18-20]. These four cells (Figure 1B and 1C) lie at the anterior margin of the blastopore and not only exhibit the typical morphology of PGCs with a large nucleus, cytoplasmic granules and a small, roundish cell shape, but also express the Vasa protein [1,18]. Thus the question arises whether these four cells constitute indeed the multipotent precursors of the MPGZ, from which the PGCs will later arise by epigenesis [1,18], or rather already the definitive PGCs.

The fate of the putative PGCs in *Platynerei*s cannot be traced directly during gastrulation by immunohistochemistry, since maternal Vasa protein decreases to background levels after 10 hpf, while zygotic Vasa protein appears only in the MPGZ from the late trochophore larva stage onwards (> 24 hpf). The transcripts of *vasa*, *nanos*, and *piwi *are initially broadly distributed and only later get progressively restricted to the MPGZ during larval development, thus *in situ *hybridization is also not suitable for monitoring the PGCs at that stage [1]. Finally, injection of lineage tracer into single blastomeres at later stages of development in *Platynereis *is extremely difficult due to small blastomere size and a tough vitelline envelope [21]. Therefore, a different approach was employed to label the PGCs: Assuming that the four secondary mesoblasts constitute the definitive four PGCs these cells will form earlier than the cells of the MPGZ [18]. Using EdU as a proliferation marker, the PGCs and the cells of the MPGZ were indeed shown to differ in their time of formation and proliferation. Furthermore, the PGCs form a distinct cluster lying at the anterior margin of the MPGZ. This challenges the assumption of PGC formation by epigenesis in the polychaete.

## Methods

### Culture and breeding of *Platynereis dumerilii*

Animals were cultured in artificial sea water (ASW) as described in [1].

### Double-labeling for Vasa expression and cell proliferation

Embryos were dejellied by rinsing on a sieve of 15 μm mesh size with 0.5 l of ASW. Incubation with 0.5-2 μM 5-ethynyl-2'-deoxyuridine (Invitrogen) [22] was carried out in six well plates in ASW for different periods ranging from 1 hpf up to 7 days post fertilization (dpf). Subsequently, the specimens were washed thoroughly in ASW and allowed to develop further until the desired stage was reached. Specimens were then fixed in 4% PFA/2xPBT and immunohistochemistry for Vasa protein was performed using affinity purified polyclonal anti-Vasa antibodies as described previously [1]. EdU incorporated during S-phase of mitosis was detected following immunohistochemistry using the click-it EdU Alexa Fluor 594 Imaging Kit (Invitrogen) according to the manufacturer's instructions. Specimens were mounted in DABCO glycerol and images were taken on a Nikon A1R confocal laser scanning microscope with a Plan Apo VC 20x NA 0.75 objective.

## Results and discussion

### The primordial PGCs in *Platynereis *arise early in development

**Figure 2:**
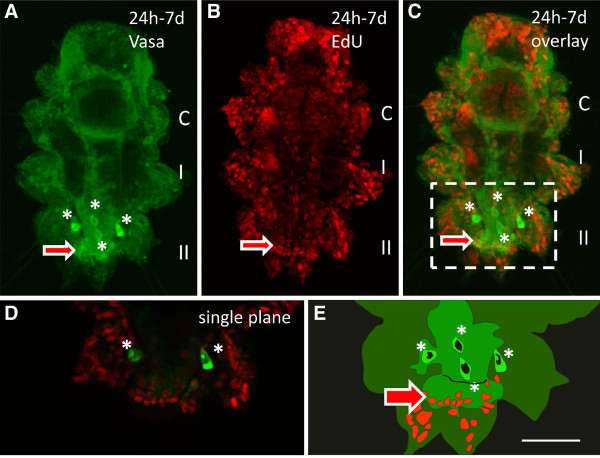
**The PGCs do not form during larval development**. Larvae were incubated 24 hpf to 7 dpf in EdU and fixed for Vasa protein and EdU detection at 7 dpf. (**A**) Vasa protein (green) is detectable in the four PGCs (asterisks) and the MPGZ (arrow). (**B**) Nuclei of proliferating cells have incorporated EdU (red) during development. (**C**) Overlay. While the cells of the MPGZ exhibit EdU labeled nuclei (arrow), the four PGCs have not divided during the incubation period (asterisks). (**D**) Single plane image showing more clearly that the nuclei of the PGCs (asterisks) are EdU negative. Note that the other two PGCs lie in a different focal plane. (**E**) Schematic drawing of the corresponding region in (C) (dashed box). The three larval segments are labeled C = cephalic segment, I, and II. Anterior is to the top. Images are merged stacks of confocal images, except the single plane image in (D). Scale bar corresponds to 50 μm in A-C, and 20 μm in D and E.

**Figure 3:**
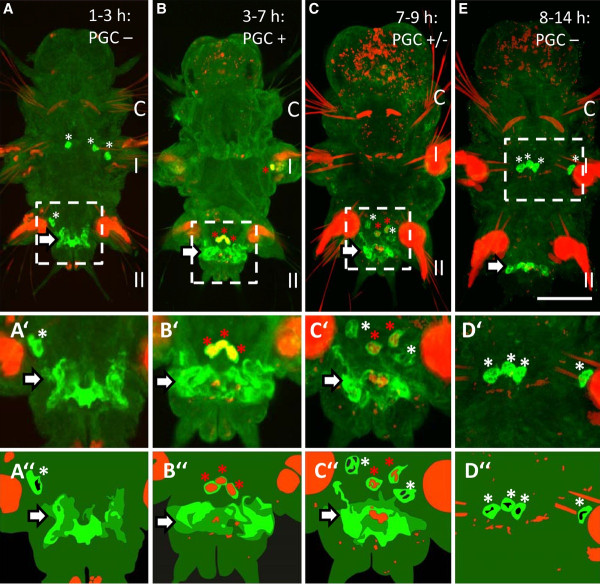
**The PGCs arise between 3-8 hpf**. Embryos were incubated for different periods in EdU and the developing young worms were fixed for Vasa and EdU detection at at 7 dpf. (**A**) Neither PGCs nor the MPGZ have incorporated EdU between 1 and 3 hpf. (**B**) Nuclei of the PGCs, but not of the MPGZ, are labeled at 3-7 hpf. (**C**) When embryos were incubated from 7-9 hpf, only two out of four PGCs are labeled. (**D**) No labeled PGCs are detectable, when embryos were incubated later than 8 hpf. Asterisks: PGCs, red: incorporation of EdU, arrows: MPGZ, green: Vasa immunohistochemistry as a marker for PGCs and the MPGZ. A-D are overlayed stacks of confocal images. A'-D' show the PGCs at higher magnification. A"-D" are schematic representations. The three larval segments are labeled C = cephalic segment, I, and II. Anterior of the larvae is to the top. Scale bar = 50 μm in A-D and 20 μm in A'-D'.

The four PGCs of *Platynereis *are first detectable at 96 hpf, when they leave the MPGZ and start to migrate anteriorly towards the primary gonad. Later they form gonial clusters which enter the coelomic cavity and finally develop into germ cells [1]. However, larvae incubated with EdU between 24 hpf and 7 dpf of development do not exhibit any labeled nuclei in the PGCs (Figure 2) suggesting that the PGCs rather have formed prior to the trochophore stage. In order to further narrow down the moment of PGC formation, embryos were incubated for shorter periods and the findings were correlated to the known cell lineage of *Platynereis *[20,23]. Here, it has to be taken into account that the incorporation of the thymidine analogon EdU occurs during the S-phase of mitosis [22] and therefore has to be applied prior to the division of the cell of interest, for a period depending on the length of the cell cycle. In *Platynereis *embryos, cell cycle duration increases from around 30 min in the first four cleavage rounds to 45 to 85 min in the fifth cleavage, demarcating the putative midblastula transition [20]. In this study, incubation starting 30 min prior to the division for at least 1 h was found sufficient to reliably label the cells of interest. During the first 3 h of development, thus prior to the formation of the mesoblast 4d, EdU is not detectable in the PGCs at 7 dpf (Figure 3A). Instead, all four PGCs exhibit EdU-positive nuclei under the same conditions, when the embryos were incubated for 3-7 hpf (Figure 3B). This period spans the formation of the mesoblast, the two primary mesoblasts, and the subsequent budding of the first pair of 'secondary mesoblasts' at 6.5 hpf (Figure 1B and Additional file 1: Figure S1). The second pair of secondary mesoblasts, which form slightly later at around 7.5 hpf, still has time to incorporate EdU during the preceding S-phase and is therefore labeled as well. Even an incubation of 6-8 hpf was still sufficient to label all four PGCs (see Additional file 2: Figure S2). Instead, when embryos were incubated between 7 and 9 hpf, thus after formation of the first but during formation of the second pair of secondary mesoblasts, only two out of four PGCs are EdU-positive at 7 dpf (Figure 3C). Embryos incubated with EdU after the formation of the second pair of secondary mesoblasts (8-14 hpf or later) lack EdU-labeled PGCs completely (Figure 3D). These results strengthen the hypothesis posed by Schneider and Bowerman 2007, that the secondary mesoblasts, which form at 6.5 and 7.5 hpf, respectively, indeed already constitute the definitive PGCs of *Platynereis*, rather than the founder cells of the MPGZ [1,18] or mesendoblasts [20,23]. An early formation of PGCs directly from the 4d lineage in the trochophore stage has also been described previously in the polychate *Salmacia dysteri *[24]. In the oligochaete *Tubifex*, *vasa *expressing cells derived from the 4d lineage are detectable in the ventral germband from the gastrulation stage onward [25]. In the polychaete *Capitella telata*, PGCs are already detectable in the early trochophore (stage 5) as two *piwi*, *vasa*, *and nanos*-expressing cell clusters in the mid-body region, while the posterior growth zone forms later at stage 6 in a more posterior region [17,26]. As in *Platynereis*, the PGCs of *Capitella *do not divide during larval development, as judged by EdU incoporation experiments [26]. In this species however, the development of the PGCs between the formation of 4d and the time, when PGCs get first detectable in larval stages, remains elusive. Using short EdU pulses as in *Platynereis *might possibly allow studying the early events of PGC formation in other species more easily.

### The MPGZ forms during the pre-trochophore stage

**Figure 4:**
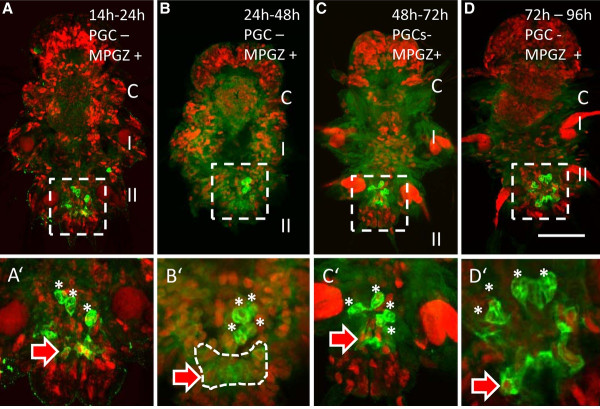
**The MPGZ starts to form at the pre-trochophore stage**. (**A**) Between 14-24 hpf, the cells of the MPGZ incorporate EdU, while the PGCs (green) lack EdU label (asterisks). (**B**) EdU positive nuclei are detectable in the MPGZ after incubation between 24-48 hpf. (**C**) EdU positive MPGZ cells are detectable in young worms incubated at 48-72 hpf. (**D**) Incubation between 72-96 hpf also yields EdU labeled nuclei in the MPGZ. A'-D': higher magnification of the MPGZ from the corresponding stages. Larvae are fixed at 6-7 dpf. EdU labeling: red. Immunohistochemistry against Vasa protein: green. Arrow and dashed line: MPGZ, asterisks, PGCs. The three larval segments are labeled C = cephalic segment, I, and II. Anterior is to the top. Images are merged stacks of confocal images. Scale bar corresponds to 50 μm in A-D, and 20 μm in A'-D'.

Incubation of early embryos with EdU (1-14 hpf, see previous section and Figure 3) fails to label the MPGZ, except for four centrally located cells which might constitute somatic descendants of the mesoblasts 4d^1 ^and 4d^2 ^(Figure 4A and Additional file 1: Figure S1). In contrast, EdU-labelled nuclei are present in the MPGZ of larvae which were incubated at later stages of development (14-24 hpf, 24-48 hpf, 48-72 hpf, or 72-96 hpf, Figure 4B-D), suggesting that the MPGZ forms from the pre-trochophore stage [27] onward. This is in accordance with the finding, that specific expression of *vasa*, *nanos*, *piwi*, or *PL10 *in the MPGZ starts around the trochophore stage [1] and probably reflects the formation of this structure. The cells of the MPGZ maintain their proliferating behavior further throughout larval stages. In the polychaete *Capitella*, the MPGZ forms in stage 6 of development, while the PGCs are already detectable in a more anterior region as early as in stage 5 [26]. A similar condition is found in the chordate *Branchiostoma*, where formation of the PGCs precedes the formation of the growth zone [16]. Thus, while PGCs and the cells of the growth zone in *Platynereis *express a similar set of stem cell specific genes, they nevertheless constitute distinct cell populations differing in their time of formation as well as their future fate.

### The cluster of PGCs lies anterior of the MPGZ

**Figure 5:**
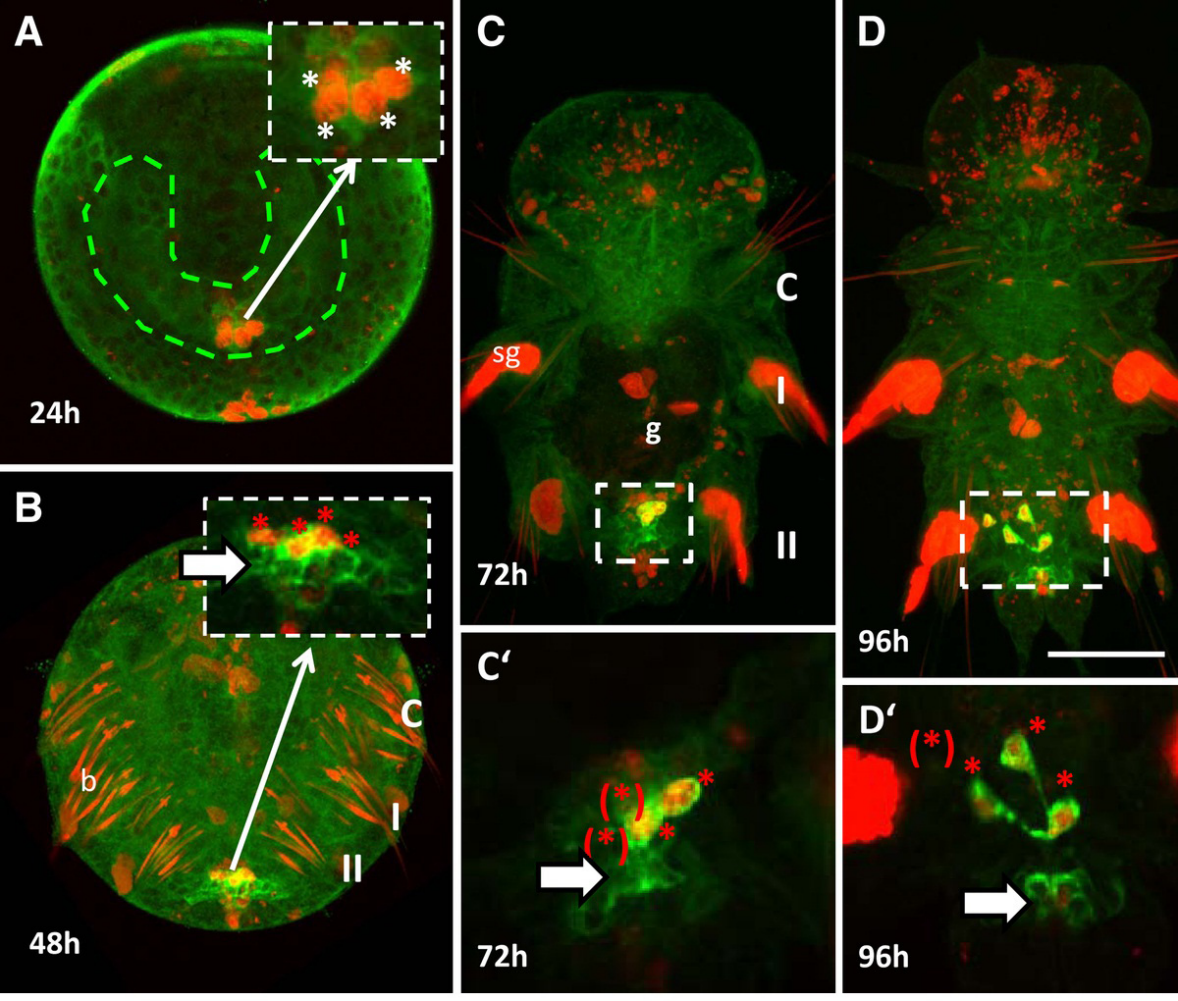
**The four PGCs form a cluster anterior to the MPGZ**. (**A**) In the trochophore larva (24 hpf), four EdU-positive PGCs are located postero-ventral at the basis of the mesodermal bands. Inset: single plane image of the posterior region in B at higher magnification (**B**) PGCs double labeled with anti-Vasa antibody (green) and EdU (red) are positioned anterior to the Vasa-positive MPGZ in the metatrochophore larva (48 hpf). Inset: Single plane image of the posterior region in B at higher magnification. (C) At 72 hpf, the four double-labeled PGCs are still forming a cluster anterior to the Vasa positive MPGZ. In the developing gut, the large EdU-positive nuclei of the four macromeres are visible. C': single plane image at higher magnification, two PGCs are located at a different focal plane (asterisk). (D) At 96 hpf, four single PGCs leave the MPGZ and start migrating anteriorly towards the primary gonad. D': Single plane image at higher magnification. One of the PGCs lies at a different focal plane (asterisk). Cells which incorporated EdU between 3-7 hpf exhibit red fluorescence in the nuclei. Bristles (b) and spinning glands (sg) are non-specifically stained by the EdU-detection reagent. Green: Vasa expression, asterisks: PGCs, red: incorporation of EdU, arrow: MPGZ, green dashed line: mesodermal bands, g = gut. The three larval segments are labeled C = cephalic segment, I, and II. Anterior of the larvae is to the top. A-D are overlays of confocal images. Insets C'-D' are single focal plane images, therefore not all the structures are always visible. Scale bar = 50 μm in A-D and 20 μm in C'-D'.

Both PGCs and the cells of the MPGZ of *Platynereis *were up to now indistinguishable in terms of morphology and gene expression profile [1]. In order to visualize the PGCs within the MPGZ, embryos were incubated with EdU from 3-7 hpf, which had been previously shown to be sufficient to label them specifically (Figure 3B). Then, larvae were fixed at 24, 48, 72, or 96 hpf, respectively. In the 24 hpf trochophore larva, four EdU positive cells can be found at the base of the mesodermal bands (Figure 5A). Vasa protein is not detectable in this stage, which is in accordance with previous findings [1]. Vasa-positive cells become visible by Vasa immunofluorescence in the MPGZ of the metatrochophore larva (48 hpf). Notably, these cells lie posterior to the group of four EdU positive PGCs (Figure 5B). Similarly, the four EdU positive PGCs form a distinct cluster in the 72 hpf nectochaete larva (Figure 5C). One day later, the PGCs start their migration towards the anterior part of the young worm (Figure 5D). Thus, the PGCs can be detected as a distinct, anterior cell population with this method even within the MPGZ.

## Conclusion: the PGCs in *Platynereis *emerge early in development by preformation

**Figure 6:**
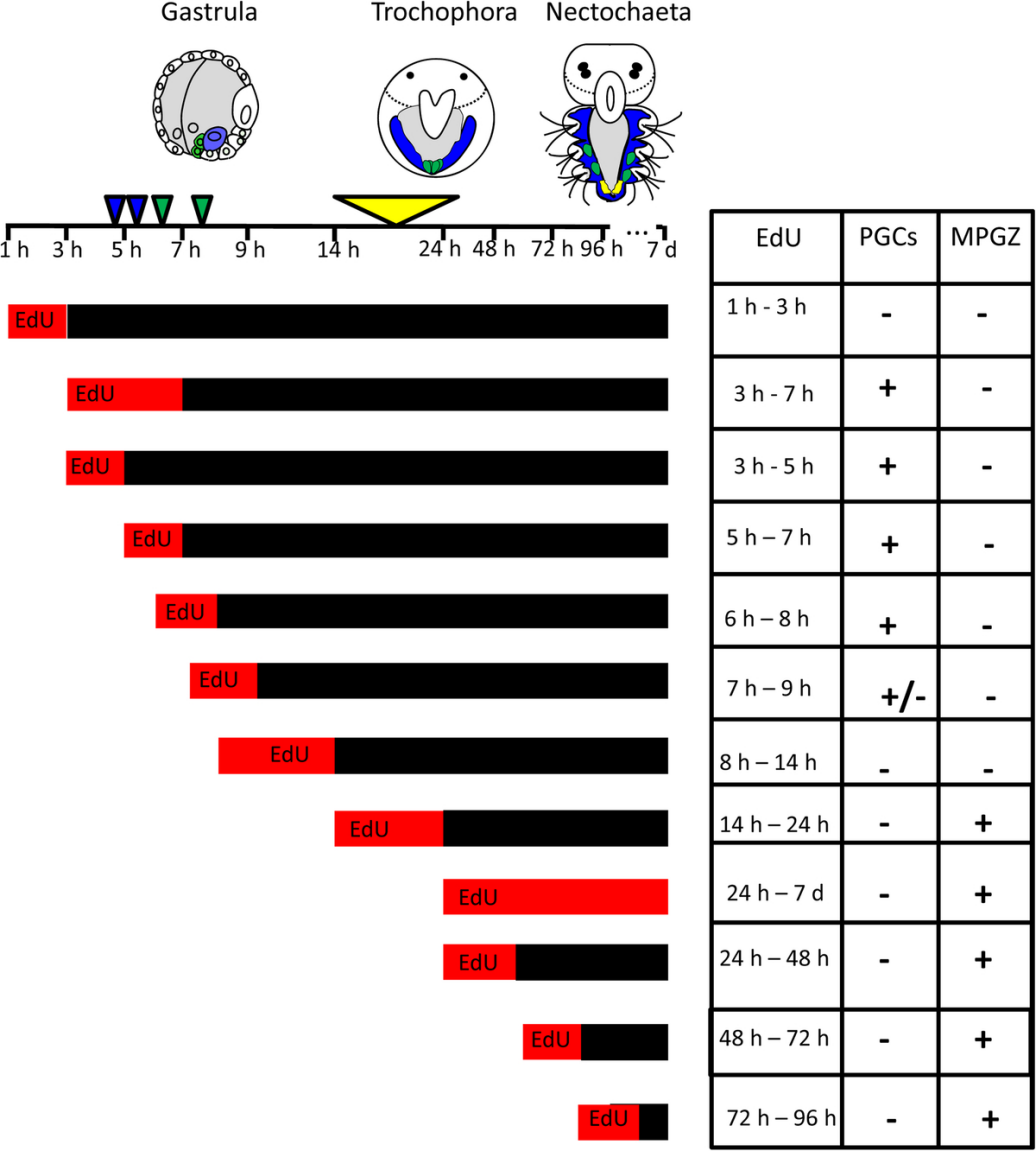
**Incorporation of EdU in PGCs and MPGZ**. Treatment with EdU for 3-8 hpf yields four labeled PGCs. Incubation during the emergence of the second pair of PGCs (7-9 hpf) lead to partial labeling of the PGCs, while none of the PGCs is labeled when incubation occurred after 8 hpf. Note: incorporation of EdU during S-phase precedes cell division by around 30 min. The MPGZ is labeled when larvae at 14 hpf or older are incubated with EdU. Arrows indicate formation of mesoblasts (blue), PGCs (green), and MPGZ (yellow), respectively. Red bars show time of EdU treatment with respect to developmental stage.

*Platynereis *belongs to the inequally cleaving spiralians [18,28], which generally exhibit an early cell fate specification by inheritance of maternal determinants, while equally cleaving spiralians rather rely on inductive processes during development [29,30]. In inequally cleaving spiralians, such as the polychaete *Capitella*, the snail *Ilyanassa*, the oyster *Crassostrea*, or the clam *Sphaerium*, putative maternal germ plasm compounds such as *vasa*, *piwi*, or *nanos *transcripts or the mitochondrial cloud enrich in the 4d lineage and subsequently in the PGCs, suggesting an early PGC formation by preformation in these species [17,31-34]. Similarly in *Platynereis*, the D-lineage, from which the PGCs arise, inherits the major proportion of Vasa protein containing yolk-free cytoplasm [1,20]. Vasa protein is then specifically enriched in the four secondary mesoblasts prior to gastrulation (Figure 1C). Pulse labeling of the secondary mesoblasts by EdU between 6 and 8 hpf later yields four EdU positive PGCs in 7-day-old young worms (Figure 3B, Additional file 2: Figure S2, summarized in Figure 6). We therefore propose that the PGCs in *Platynereis *are formed by preformation early in development by inheritance of a Vasa protein containing germ plasm, rather than by epigenesis from the MPGZ. The cells of the MPGZ instead arise later in the pre-trochophore stage (Figures 4 and 6) and lie posterior to the cluster of PGCs (Figure 5B). Our data suggest that only the four primordial PGCs located anterior of the MPGZ contribute to the germline in *Platynereis*.

## Abbreviations

Dpf: days post fertilization; EdU: 5-ethynyl-2'-deoxyuridine; hpf: hours post fertilization; MPGZ: mesodermal posterior growth zone; PGC: primordial germ cell.

## Competing interests

The authors declare that they have no competing interests.

## Authors' contributions

NR designed and coordinated the study, carried out most of the experiments, analyzed most of the data, and drafted the manuscript. AL carried out some of the experiments, analyzed some of the data, and helped to draft the manuscript. CFA performed the imaging and helped to draft the manuscript. All authors read and approved the final manuscript.

## Supplementary Material

Additional file 1 **Figure S1**. Cell lineage of the D-Quadrant. The micromere 4d divides bilaterally, yielding a pair of mesoblasts (4d^1 ^and 4d^2^). These mesoblasts (blue) bud of two pairs of secondary mesoblasts (green), the cells 4d^12^, 4d^111^, 4d^22^, and 4d^212^. Time of formation is indicated in red. Macromeres are shown in grey. Cell lineage modified after [19,20]. Click here for file

Additional file 2 **Figure S2**. The PGCs form between 6 and 8 hours post fertilization (hpf). Larvae were incubated between 6 and 8 hpf in EdU and fixed for Vasa protein and EdU detection at 7 dpf. (**A**) Vasa protein (green) is detectable in the four PGCs (asterisks) and the MPGZ (arrow). (**B**) Cells which proliferated between 6 and 8 hpf have incorporated EdU (red) during development, including the four PGCs (asterisks). (**C**) Overlay: The four Vasa positive PGCs exhibit EdU labeled nuclei. The three larval segments are labeled C, I, and II. Anterior is to the top. Scale bar corresponds to 50 μm. Click here for file
