# Supplementary figures and images for: Hidden in the crowd: primordial germ cells and somatic stem cells in the mesodermal posterior growth zone of the polychaete Platynereis dumerillii are two distinct cell populations
Source: EvoDevo. 2012 Apr 18;3:9. doi: 10.1186/2041-9139-3-9 (PMC3348064; doi:10.1186/2041-9139-3-9)

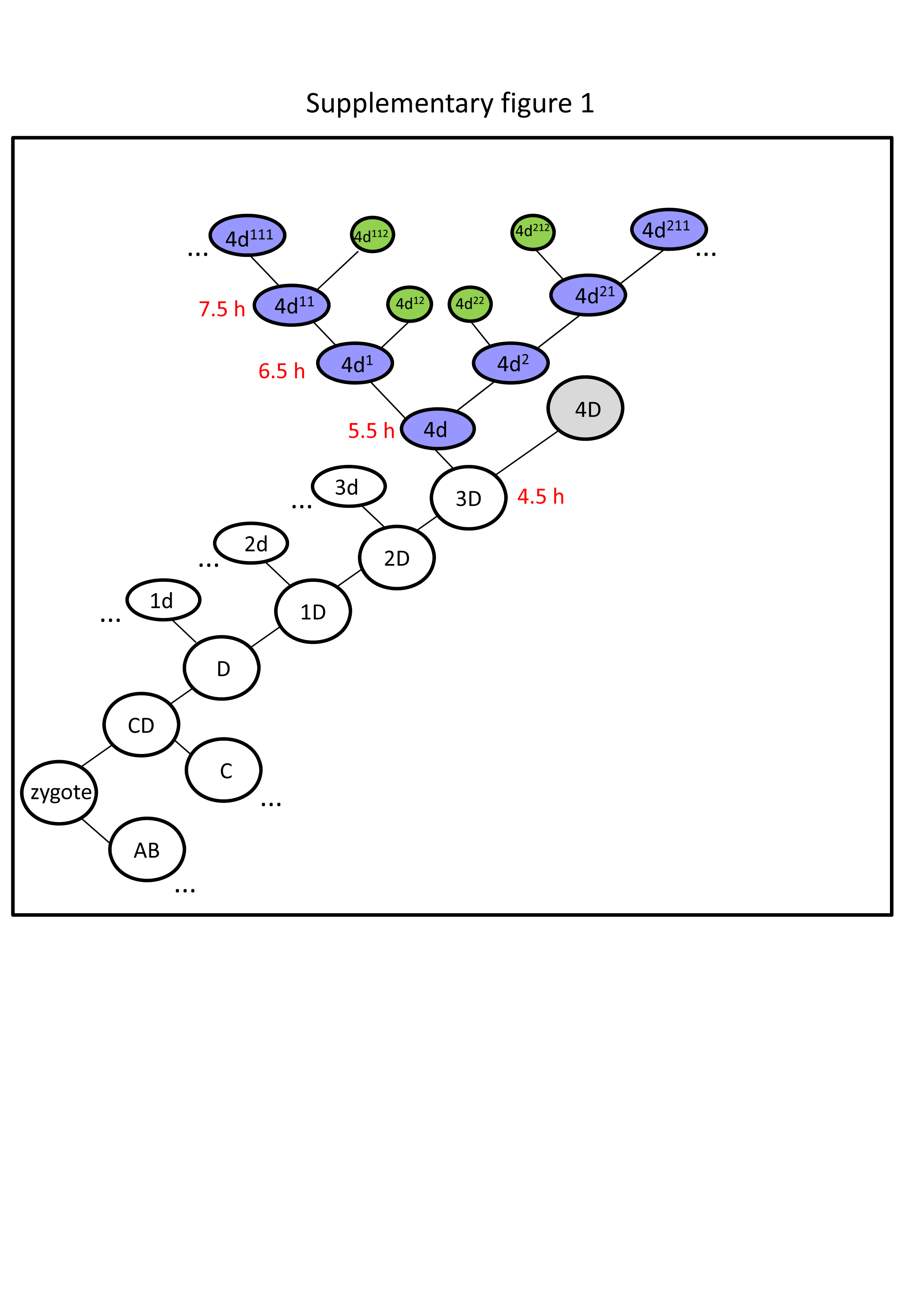

Supplement: Additional file 1 — Figure S1. Cell lineage of the D-Quadrant. The micromere 4d divides bilaterally, yielding a pair of mesoblasts (4d1 and 4d2). These mesoblasts (blue) bud of two pairs of secondary mesoblasts (green), the cells 4d12, 4d111, 4d22, and 4d212. Time of formation is indicated in red. Macromeres are shown in grey. Cell lineage modified after [19,20]. [file 2041-9139-3-9-S1.TIFF]

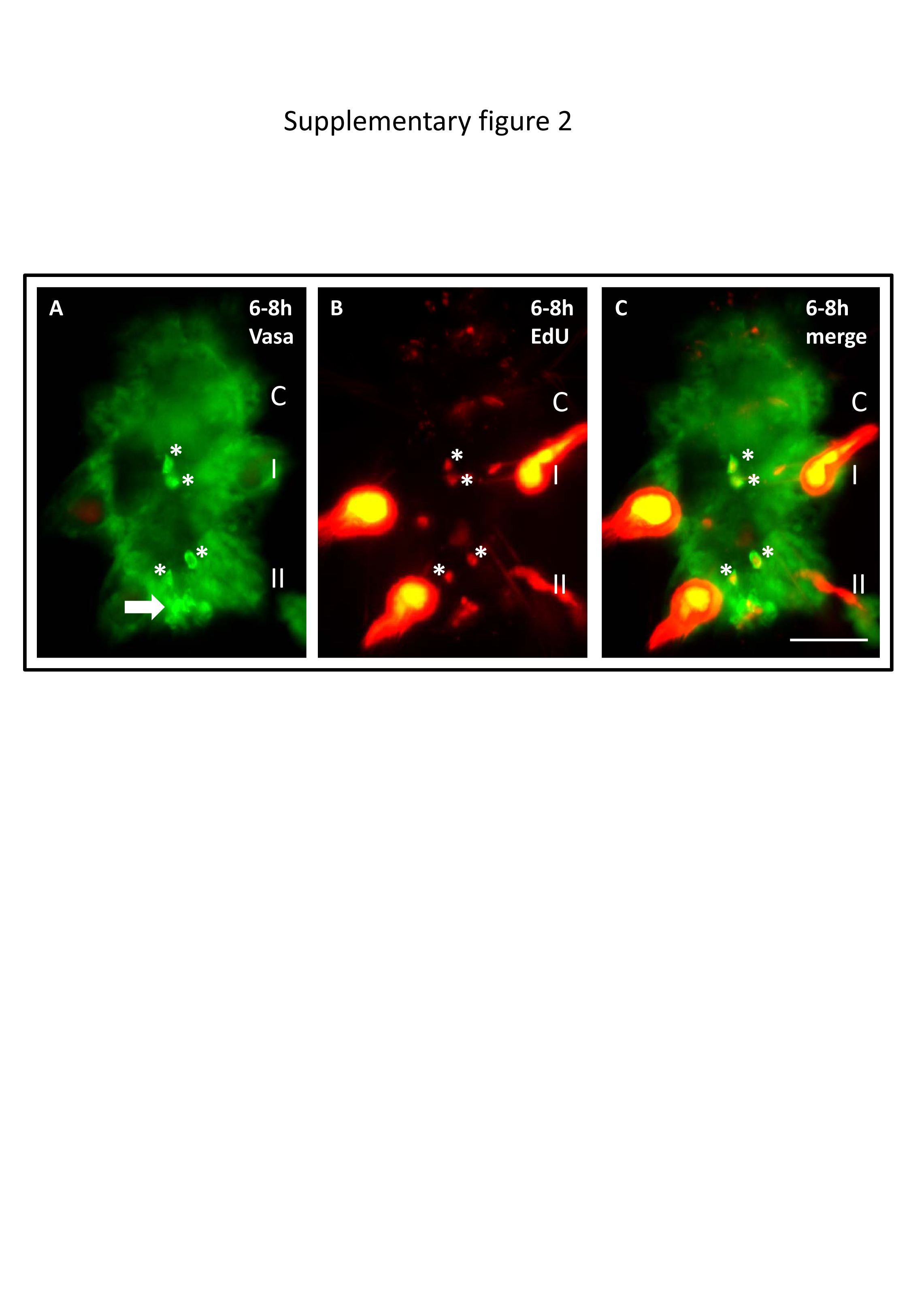

Supplement: Additional file 2 — Figure S2. The PGCs form between 6 and 8 hours post fertilization (hpf). Larvae were incubated between 6 and 8 hpf in EdU and fixed for Vasa protein and EdU detection at 7 dpf. (A) Vasa protein (green) is detectable in the four PGCs (asterisks) and the MPGZ (arrow). (B) Cells which proliferated between 6 and 8 hpf have incorporated EdU (red) during development, including the four PGCs (asterisks). (C) Overlay: The four Vasa positive PGCs exhibit EdU labeled nuclei. The three larval segments are labeled C, I, and II. Anterior is to the top. Scale bar corresponds to 50 μm. [file 2041-9139-3-9-S2.TIFF]
